# Ultrasonic Enhancement of Drug Penetration in Solid Tumors

**DOI:** 10.3389/fonc.2013.00204

**Published:** 2013-08-19

**Authors:** Chun-Yen Lai, Brett Z. Fite, Katherine W. Ferrara

**Affiliations:** ^1^Department of Biomedical Engineering, University of California Davis, Davis, CA, USA

**Keywords:** ultrasound, sonoporation, vascular permeability, tumor penetration, enhanced drug delivery

## Abstract

Increasing the penetration of drugs within solid tumors can be accomplished through multiple ultrasound-mediated mechanisms. The application of ultrasound can directly change the structure or physiology of tissues or can induce changes in a drug or vehicle in order to enhance delivery and efficacy. With each ultrasonic pulse, a fraction of the energy in the propagating wave is absorbed by tissue and results in local heating. When ultrasound is applied to achieve mild hyperthermia, the thermal effects are associated with an increase in perfusion or the release of a drug from a temperature-sensitive vehicle. Higher ultrasound intensities locally ablate tissue and result in increased drug accumulation surrounding the ablated region of interest. Further, the mechanical displacement induced by the ultrasound pulse can result in the nucleation, growth and collapse of gas bubbles. As a result of such cavitation, the permeability of a vessel wall or cell membrane can be increased. Finally, the radiation pressure of the propagating pulse can translate particles or tissues. In this perspective, we will review recent progress in ultrasound-mediated tumor delivery and the opportunities for clinical translation.

## The Problem

The goal of this Frontiers issue is to explore methods to enhance the penetration of drugs within solid tumors. Combining ultrasound with a drug does indeed have the potential to enhance delivery; however, due to the requirement to guide the beam to the tumor such treatment will be a possibility only for localized primary tumors and well-characterized metastases that are accessible to sound waves. Ultrasound is easily directed to superficial organs such as the breast and prostate, as well as most abdominal organs, and has also been applied in the treatment of brain tumors. The effects of high intensity ultrasound on biological tissue, and particularly on the central nervous system, have been recognized for more than 70 years; the ability to heat and ablate tissue was described initially (1–5). Within studies in the 1940s and 1950s, ultrasound was also determined to have non-thermal effects on tissue, typically characterized as mechanical effects (6). The mechanical effects of ultrasound can act directly upon the tumor tissue or on injected microbubbles whose oscillations enhance vascular or cell membrane permeability. Although early studies were not geared toward drug delivery, these same mechanisms of high temperature ablation or mild hyperthermia can increase drug accumulation within a lesion and lesion boundary. In recent strategies, the increased temperature is applied to influence both the tissue and the drug capsule.

## Enhanced Extravasation of Nanotherapeutics through Mechanical and Thermal Effects on Tissue

The direct effects of ultrasound on tissue and vasculature have been reported to enhance the extravasation of antibodies and nanotherapeutics (7–9). In some cases, the mechanical effects of ultrasound have been shown to enhance therapeutic penetration. With a center frequency of 1 MHz ultrasound at a peak negative pressure (PNP) of 8.95 MPa, antibody penetration has been shown to be enhanced at the tumor periphery, presumably through mechanical effects (8). The compression and rarefaction resulting from the ultrasound wave can produce the nucleation, growth, and collapse of gas bubbles within tissues. Such cavitation is assumed to facilitate transport within tumor tissue.

The thermal dose delivered by ultrasound is typically measured in cumulative equivalent minutes at 43°C (CEM 43) which is defined as *tR*^(43 − *T*)^, with *t* being the time of treatment, *T* the average temperature during treatment, and *R* a constant that equals 0.25 for temperatures between 37 and 43°C and 0.5 above 43°C (10, 11). Hyperthermia has been demonstrated to increase tumor blood flow and microvascular permeability (12). While it has long been recognized that heat increases the accumulation of small particles in the heated region of interest, the typical protocol has involved 1 h or more of heating. However, by combining the mechanical and thermal effects of ultrasound, enhanced delivery has been achieved with a shorter treatment (13). In such studies, the temperature goal is ∼41–42°C and insonation continued for ∼5–20 min. As a result of hyperthermia and the mechanical effects of ultrasound, we have observed that the accumulation of liposomes in an insonified tumor can be increased up to threefold to as much as 22%ID/g. While ultrasound was shown to enhance accumulation in syngeneic murine tumors, the ultrasound parameters that were required to enhance nanoparticle accumulation were shown to differ between epithelial and epithelial-mesenchymal transition (EMT) tumor phenotypes (7). While mild hyperthermia enhanced accumulation in the epithelial tumors, likely through decreased intratumoral pressure and enhanced apparent permeability, higher ultrasound pressure was required to enhance delivery in the poorly vascularized EMT phenotype. Further, excessive temperature or thermal dose can result in vascular stasis, particularly in highly vascular epithelial tumors. The requirement to personalize the ultrasound parameters to the tumor biology will likely require image guidance to insure clinical success.

In part due to the differing effects of mild hyperthermia with tumor biology, the use of high temperature ablation to enhance delivery has been explored as a methodology that is likely to be generally effective in increasing delivery. While it seems counterintuitive that tissue ablation can greatly enhance accumulation, edema, enhanced blood flow, and increased transport in the region surrounding the ablated site can successfully improve delivery. In our experience, the peak delivery in regions surrounding ablation can exceed 30%ID/g. Also of clinical interest, the hyperthermia surrounding radiofrequency (rf) ablation lesions has been used to enhance local delivery; however, the temperature obtained with such devices ranges from 50 to 90°C (14). Rf ablation has been applied in previous studies to achieve a similar enhanced delivery, and such techniques are now in clinical trials (15, 16). High intensity focused ultrasound similarly enhances delivery surrounding the site of ablation, although combinations of ablation and drug delivery remain primarily under pre-clinical investigation.

## Release of Drug from Nanoparticles within the Vasculature

Nanoparticles that can be triggered to release a small molecule cargo within a tumor have shown the potential to increase both the local concentration of the drug and tumor penetration. Yet, the challenge of developing particles that are stable in circulation and release their cargo upon activation has long been recognized as a major challenge in pharmaceutical development. While many activatable particles are under development (11, 17–23), thermally sensitive liposomes have been frequently combined with ultrasound in recent pre-clinical and clinical studies and will be considered here. In studies of thermally sensitive liposomes, imaging has been used to verify that amphipathic cargo released within the tumor vasculature remains concentrated within the tumor in the region of release (18). We have found that release of drug from such temperature-sensitive vehicles can be highly effective, resulting in a complete response in aggressive murine tumors (unpublished data).

Temperature-sensitive liposomes were initially proposed containing 1,2-dipalmitoyl-*sn*-glycero-3-phosphocholine (DPPC) with a phase transition of *T*_m_ = ∼41°C and multiple formulations containing DPPC have been proposed (24, 25). The incorporation of lyso-phospholipids in DPPC-based liposomes decreases the phase transition temperature and speeds the release of the cargo, likely due to the creation of local defects within the lipid bilayer (26). The Thermodox™ formulation, with the incorporation of a lyso*-*phospholipid, releases at a clinically desirable temperature of ∼39°C. The incorporation of the lyso*-*phospholipids also enhances the ion permeability and drug release rates at the membrane phase transition (27). Unfortunately, using the conventional ammonium sulfate loading, liposomes containing lyso*-*phospholipids also rapidly release their cargo within the blood pool. As a result, while local delivery can be achieved within tens of minutes after injection, the dose limiting toxicities of such formulations have typically limited their application to single dose administration. Although the pre-clinical data using Thermodox has been very exciting, this activatable doxorubicin formulation reportedly failed to meet its primary endpoint in the Phase III HEAT Study in patients with hepatocellular carcinoma (HCC). Yet, in spite of this setback, the potential for temperature-sensitive vehicles to have a significant impact on the concentration and penetration of drugs within solid tumors is substantial. Although early clinical studies have typically been limited to one-time treatment, with new formulations repeated treatment should be feasible and the resulting clinical impact enhanced. Multiple alternative formulations have been proposed and compared and have been shown to enhance circulation time (25, 28–30). Alternative strategies using metal-drug complexes, a Brij surfactant and phosphatidylglycerol have been reported to enhance the stability of temperature-sensitive liposomes and are promising alternatives for future investigation. The ultrasound parameters used to enhance delivery with temperature-sensitive liposomes have also varied widely with the center frequency typically ranging from 1 to 3 MHz and duty cycle ranging from ∼10 to 100% (20, 31, 32).

## Microbubbles

Micron-scale gas bubbles with a stabilizing shell are used in ultrasound imaging to improve imaging of the blood pool and have been widely applied in pre-clinical studies of enhanced drug delivery. The microbubble shell can be coupled to nanotherapeutics, such as liposomes, or coated with a drug (33, 34). The gas core can transport oxygen or other useful gas cargo, although for imaging the gas core is selected to reduce diffusion through the shell material (35). Alternative formulations in which liquid perfluorocarbon particles are injected and change to a gaseous phase *in vivo* have also been shown to have efficacy in the delivery of drugs to solid tumors (36).

Reflections of ultrasound waves from tissue increase in proportion to variations in density and compressibility of the medium and therefore highly compressible gas bubbles produce strong ultrasound echoes. These small bubbles expand and contract in response to ultrasound waves. When driven at a frequency near the resonance frequency that is determined by the size and physical composition of the microbubble, a multi-fold expansion can result. During the subsequent collapse, the velocity of the microbubble wall can reach hundreds of meters per second (37, 38), and the gas core can fragment into a set of small gas particles (39). Also, during microbubble collapse, small jets can impact nearby cell membranes and result in enhanced transport of materials into the cell. *In vitro* studies in phantom materials and *ex vivo* studies within tissues have confirmed that the oscillating microbubble can travel through the vessel wall or can affect the mechanical integrity of the vessel (40–42). Still, such jets affect cells only within a distance on the order of tens of microns. Therefore, the application of microbubbles to alter vascular, rather than tumor cell, permeability is attractive since the vascular concentration is initially high and large numbers of microbubbles are required to effectively change the membrane permeability of a large fraction of cells within a tissue. Within the vasculature, catheters have also been applied to direct streams of bubbles to a region of interest (43). Also, microbubble-enhanced gene delivery has been widely studied since the biological amplification resulting from transfection is expected to increase the impact of treatment although the protocols have varied (44–48). Within such studies, microbubbles and DNA have been co-injected or combined into a single vehicle and the administration has been intratumoral or intravascular. Typical ultrasound parameters for enhanced tumor gene delivery have included a center frequency on the order of 1 MHz and a low pulse repetition frequency; however, the peak negative ultrasound pressure has varied significantly and successful transfection at higher ultrasound frequencies has also been reported.

The ultrasound parameters used to increase vascular permeability must be chosen carefully as insonation of microbubbles with low ultrasound frequencies has been shown to reduce blood flow (49). A parameter space for safe and effective use of microbubbles for enhancing vascular permeability has been established (41, 50–52). Many parameters, including microbubble dosage and size, the ultrasound center frequency, pulse duration, pulse repetition frequency, and the PNP determine the effect of the oscillating microbubble on the surrounding tissue.

In addition to the formation of jets, physical mechanisms exploited in microbubble-enhanced delivery include radiation forces and microstreaming of fluid (53, 54). Radiation force refers to a mechanism by which oscillating microbubbles or other particles are displaced, most typically in the direction of wave propagation (55–57). This displacement can be used to enhance vascular targeting of a microbubble or microbubble-drug conjugate. Further, local motion of fluid surrounding the oscillating bubble is known as microstreaming and has been shown to increase cellular uptake of therapeutics (53, 54).

One of the most important applications for the use of microbubbles to enhance delivery has been the enhancement of blood brain barrier (BBB) permeability (58–65). In general, the technique consists of systemically injecting microbubbles and insonifying the region of the brain where enhanced permeability is desired. Here again, a parameter space has been established within which enhanced BBB transport is achieved with minimal hemorrhage and cell death. Still, the extension of these techniques into the clinic is expected to require the use of real-time cavitation detection, as individual variations in the skull penetration of the ultrasound wave are significant and the therapeutic window for effective and safe therapy is relatively small (66). As noted above, pre-clinical-application of microbubble-enhanced delivery is widespread. In addition, the authors are aware of yet unreported small clinical studies of microbubble-enhanced delivery to solid tumors.

## Application of Imaging Technology

A major reason for the expansion of the application of therapeutic ultrasound is the development of methods to monitor the treated location and the temperature using MRI (67) or ultrasound (68). While mild hyperthermia (CEM 43 < 0.5) is associated with increased metabolism, blood flow, and tissue repair, higher thermal doses are associated with enhanced cell death and therefore the methods to carefully control and monitor the delivered temperature are critically important (69). Image guidance using nuclear medicine techniques is also attractive due to their high sensitivity and the opportunity for quantitation of delivery (70, 71). By radiolabeling nanoparticles, the rate and magnitude of extravasation can be directly estimated from PET data (71). Even with the relatively low spatial resolution of PET (∼1 mm), the penetration of nanoparticle-based therapeutics has been assessed and shown to differ from small molecular weight agents (72).

In order to fully evaluate the enhanced penetration of a drug resulting from ultrasound, multiple imaging labels can be incorporated with drug accumulation and penetration assessed at the whole body, organ, and cellular scales (8, 9, 70, 73, 74). Multiple MRI protocols can be proposed for the guidance of ultrasound therapies including diffusion-weighted (75, 76), T2-weighted (77, 78), and contrast enhanced T1-weighted imaging (79–81), fluid attenuated inversion recovery (FLAIR) (82), heteronuclear (^23^Na) (83), spectroscopy (84), and displacement sensitive sequences via MR elastography (85). Following HIFU ablation of the prostate, gadolinium enhanced MRI is often used to evaluate the extent of tissue damage. Although contrast enhanced T1-weighted MRI can detect tissue damage following HIFU ablation (86, 87), it does not correlate to histological results (intensity of necrosis, presence of foci of viable cancer) immediately following HIFU (87). However, for follow-up examinations, DCE MRI has demonstrated good sensitivity and diffusion MRI has shown specificity in identifying tumor progression after HIFU ablation (75, 88).

In addition to endogenous contrast mechanisms that can be used to guide and assess ultrasound therapies, exogenous agents can be used to report on specific changes. For example, co-administration of two paramagnetic contrast agents (gadolinium and thulium) within liposomal drug carriers has been previously utilized to follow internalization and cellular trafficking of the vehicle (89). Similarly, multi modal liposomal agents spanning CT and MRI have been used to assess the penetration of liposomes within tumors and have been proposed for cross modality registration and as a means to guide imaging-based interventions (73, 74). Many physiological parameters can also be assessed by MRI and coupled with the soft tissue anatomical information motivate MRI as an excellent tool for guiding thermal therapies (90, 91).

In addition to the role of MRI in the assessment of drug penetration and distribution, MR thermometry can be applied to monitor the temperature of a region during an intervention (92–100). The proton resonance frequency (PRF) of water is frequently used to detect changes in temperature (101) both because it has a thermal coefficient that is linear over a wide temperature range and, excepting adipose tissue, the PRF shift has little dependence on tissue type even following coagulation (99, 102). The PRF shift can be measured rapidly with gradient echo sequences, which is advantageous during thermal therapies where high temporal resolution is desirable, especially during ablative processes, to avoid damage to surrounding tissue. Further increases in temporal resolution can be gained via partial parallel imaging techniques using phased arrays (103–105) utilizing various algorithms (105).

Neither clinical focused ultrasound (FUS) systems, which typically operate around 1 MHz (106, 107), nor clinical MR scanners (e.g., 1.5, or 3 T) are ideal for small animal imaging. In the former, the focal spot depth may encompass an appreciable portion of the animal, while the latter may have insufficient signal-to-noise ratio (SNR) to easily obtain detailed images of murine tumors. The smaller focal depth at higher FUS frequencies makes them more suitable for pre-clinical imaging of small animals (108). High field scanners are especially useful for small animal thermal imaging because in addition to providing higher SNR, which can be used for higher spatial resolutions, they also improve the sensitivity of thermal measurements made with the PRF shift method, which itself has a first order dependence on magnetic field strength.

## Future Applications

The use of the thermal and mechanical effects of ultrasound to enhance delivery to solid tumors is expanding. With the increasing availability of MRI-guided high intensity focused ultrasound, well-controlled and calibrated clinical studies are feasible. Both the use of ultrasound to alter tissue properties and to release a drug from a carrier are in widespread pre-clinical evaluation. With the addition of microbubbles, drug penetration through the endothelium can also be increased, although the protocols are currently more complex due to the need to co-inject the therapeutic and microbubbles.

In the future, the effects of ultrasound may transcend the local effect through enhanced immune response. The addition of immunotherapy to standard-of-care cancer therapies has shown evidence of efficacy in the pre-clinical and clinical settings (109–115). The immune system is often tolerant to antigens presented by the tumor and therefore strategies to induce tumor-specific immunity must overcome obstacles including: insufficient and dysfunctional populations of antigen-presenting cells and lymphocytes, the difficulty of inducing potent immunity without inducing unacceptable autoimmune toxicities, the low immunogenicity of antigens expressed by tumor cells, and immunoregulatory pathways that dampen the tumor-specific immune response (116). The use of ultrasound ablation to generate an immune response has been shown to be a promising technique for immune activation (110–112, 114, 115, 117–121). Ultrasound ablation is thought to act through dendritic cell maturation and T-cell immunity (122), and is particularly advantageous because it is completely non-invasive, can be controlled with high spatial precision and uses no harmful ionizing radiation (123, 124).

## Conflict of Interest Statement

The authors declare that the research was conducted in the absence of any commercial or financial relationships that could be construed as a potential conflict of interest.

## References

[1] Wall PD Fry WJ Stephens R Tucker D Lettvin JY Changes produced in the central nervous system by ultrasound. Science (1951) 114:686–7 10.1126/science.114.2974.686 14913137

[2] Fry WJ Dunn F Ultrasonic irradiation of the central nervous system at high sound levels. J Acoust Soc Am (1956) 28:129–31 10.1121/1.1908200

[3] Fry FJ Ades HW Fry WJ Production of reversible changes in the central nervous system by ultrasound. Science (1958) 127:83–4 10.1126/science.127.3289.83 13495483

[4] Lynn JG Zwemer RL Chick AJ Miller AE A new method for the generation and use of focused ultrasound in experimental biology. J Gen Physiol (1942) 26:179–93 10.1085/jgp.26.2.179 19873337 PMC2142058

[5] Lynn J Zwemer RL Chick AJ The biological application of focused ultrasound waves. Science (1942) 96:119–20 10.1126/science.96.2483.119 17809987

[6] Fry WJ Wulff VJ Tucker D Fry FJ Physical factors involved in ultrasonically induced changes in living systems: Identification of non-temperature effects. J Acoust Soc Am (1950) 22:867–76 10.1121/1.1906707

[7] Watson K Lai CY Qin S Kruse DE Lin YC Seo JW Ultrasound increases nanoparticle delivery by reducing intratumoral pressure and increasing transport in epithelial and epithelial-mesenchymal transition tumors. Cancer Res (2012) 72:1485–93 10.1158/0008-5472.CAN-11-3232 22282664 PMC3357123

[8] Wang S Shin IS Hancock H Jang B-S Kim H-S Lee SM Pulsed high intensity focused ultrasound increases penetration and therapeutic efficacy of monoclonal antibodies in murine xenograft tumors. J Control Release (2012) 162:218–24 10.1016/j.jconrel.2012.06.025 22732476 PMC4219504

[9] Frenkel V Ultrasound mediated delivery of drugs and genes to solid tumors. Adv Drug Deliv Rev (2008) 60:1193–208 10.1016/j.addr.2008.03.007 18474406 PMC2491332

[10] Dewhirst MW Vujaskovic Z Jones E Thrall D Re-setting the biologic rationale for thermal therapy. Int J Hyperthermia (2005) 21:779–90 10.1080/02656730500271668 16338861

[11] Jones EL Oleson JR Prosnitz LR Samulski TV Vujaskovic Z Yu DH Randomized trial of hyperthermia and radiation for superficial tumors. J Clin Oncol (2005) 23:3079–85 10.1200/JCO.2005.05.520 15860867

[12] Gaber MH Wu NZ Hong K Huang SK Dewhirst MW Papahadjopoulos D Thermosensitive liposomes: extravasation and release of contents in tumor microvascular networks. Int J Radiat Oncol Biol Phys (1996) 36:1177–87 10.1016/S0360-3016(96)00389-6 8985041

[13] Yuh EL Shulman SG Mehta SA Xie J Chen L Frenkel V Delivery of systemic chemotherapeutic agent to tumors by using focused ultrasound: study in a murine model. Radiology (2005) 234(2):431–7 10.1148/radiol.2342030889 15671000

[14] Ahmed M Monsky WE Girnun G Lukyanov A D’Ippolito G Kruskal JB Radiofrequency thermal ablation sharply increases intratumoral liposomal doxorubicin accumulation and tumor coagulation. Cancer Res (2003) 63:6327–33 14559820

[15] Goldberg SN Ahmed M Minimally invasive image-guided therapies for hepatocellular carcinoma. J Clin Gastroenterol (2002) 35:S115–29 10.1097/00004836-200211002-00008 12394215

[16] Wood BJ Poon RT Locklin JK Dreher MR Ng KK Eugeni M Phase I study of heat-deployed liposomal doxorubicin during radiofrequency ablation for hepatic malignancies. J Vasc Interv Radiol (2012) 23:248–55 10.1016/j.jvir.2011.10.018 22178041 PMC3264789

[17] Viglianti BL Abraham SA Michelich CR Yarmolenko PS MacFall JR Bally MB In vivo monitoring of tissue pharmacokinetics of liposome/drug using MRI: illustration of targeted delivery. Magn Reson Med (2004) 51:1153–62 10.1002/mrm.20074 15170835

[18] Ponce AM Viglianti BL Yu DH Yarmolenko PS Michelich CR Woo J Magnetic resonance imaging of temperature-sensitive liposome release: drug dose painting and antitumor effects. J Natl Cancer Inst (2007) 99:53–63 10.1093/jnci/djk005 17202113

[19] Kong G Anyarambhatla G Petros WP Braun RD Colvin OM Needham D Efficacy of liposomes and hyperthermia in a human tumor xenograft model: importance of triggered drug release. Cancer Res (2000) 60:6950–7 11156395

[20] Dromi S Frenkel V Luk A Traughber B Angstadt M Bur M Pulsed-high intensity focused ultrasound and low temperature sensitive liposomes for enhanced targeted drug delivery and antitumor effect. Clin Cancer Res (2007) 13:2722–7 10.1158/1078-0432.CCR-06-2443 17473205 PMC2555974

[21] Loo C Lowery A Halas N West J Drezek R Immunotargeted nanoshells for integrated cancer imaging and therapy. Nano Lett (2005) 5:709–11 10.1021/nl050127s 15826113

[22] Derfus AM von Maltzahn G Harris TJ Duza T Vecchio KS Ruoslahti E Remotely triggered release from magnetic nanoparticles. Adv Mater (2007) 19:3932–6 10.1002/adma.200700091

[23] Gannon CJ Patra CR Bhattacharya R Mukherjee P Curley SA Intracellular gold nanoparticles enhance non-invasive radiofrequency thermal destruction of human gastrointestinal cancer cells. J Nanobiotechnol (2008) 6:2 10.1186/1477-3155-6-2 18234109 PMC2276230

[24] Yatvin MB Weinstein JN Dennis WH Blumenthal R Design of liposomes for enhanced local release of drugs by hyperthermia. Science (1978) 202:1290–3 10.1126/science.364652 364652

[25] Paoli EE Kruse DE Seo JW Zhang H Kheirolomoom A Watson KD An optical and microPET assessment of thermally-sensitive liposome biodistribution in the Met-1 tumor model: Importance of formulation. J Control Release (2010) 143:13–22 10.1016/j.jconrel.2009.12.010 20006659 PMC2861564

[26] Needham D Anyarambhatla G Kong G Dewhirst MW A new temperature-sensitive liposome for use with mild hyperthermia: characterization and testing in a human tumor xenograft model. Cancer Res (2000) 60:1197–201 10728674

[27] Mills JK Needham D Lysolipid incorporation in dipalmitoylphosphatidylcholine bilayer membranes enhances the ion permeability and drug release rates at the membrane phase transition. Biochim Biophys Acta (2005) 1716:77–96 10.1016/j.bbamem.2005.08.007 16216216

[28] Tagami T Ernsting MJ Li S-D Optimization of a novel and improved thermosensitive liposome formulated with DPPC and a Brij surfactant using a robust in vitro system. J Control Release (2011) 154:290–7 10.1016/j.jconrel.2011.05.020 21640149

[29] Lindner LH Eichhorn ME Eibl H Teichert N Schmitt-Sody M Issels RD Novel temperature-sensitive liposomes with prolonged circulation time. Clin Cancer Res (2004) 10:2168–78 10.1158/1078-0432.CCR-03-0035 15041738

[30] Kheirolomoom A Mahakian LM Lai C-Y Lindfors HA Seo JW Paoli EE Copper-doxorubicin as a nanoparticle cargo retains efficacy with minimal toxicity. Mol Pharm (2010) 7:1948–58 10.1021/mp100245u 20925429 PMC3037269

[31] Partanen A Yarmolenko PS Viitala A Appanaboyina S Haemmerich D Ranjan A Mild hyperthermia with magnetic resonance-guided high-intensity focused ultrasound for applications in drug delivery. Int J Hyperthermia (2012) 28:320–36 10.3109/02656736.2012.680173 22621734 PMC7641882

[32] Staruch RM Ganguly M Tannock IF Hynynen K Chopra R Enhanced drug delivery in rabbit VX2 tumours using thermosensitive liposomes and MRI-controlled focused ultrasound hyperthermia. Int J Hyperthermia (2012) 28:776–87 10.3109/02656736.2012.736670 23153219

[33] Lum AFH Borden MA Dayton PA Kruse DE Simon SI Ferrara KW Ultrasound radiation force enables targeted deposition of model drug carriers loaded on microbubbles. J Control Release (2006) 111:128–34 10.1016/j.jconrel.2005.11.006 16380187 PMC1526414

[34] Kheirolomoom A Dayton PA Lum AFH Little E Paoli EE Zheng H Acoustically-active microbubbles conjugated to liposomes: characterization of a proposed drug delivery vehicle. J Control Release (2007) 118:275–84 10.1016/j.jconrel.2006.12.015 17300849 PMC2662343

[35] Kwan JJ Kaya M Borden MA Dayton PA Theranostic oxygen delivery using ultrasound and microbubbles. Theranostics (2012) 2:1174–84 10.7150/thno.4410 23382774 PMC3563146

[36] Rapoport N Phase-shift, stimuli-responsive perfluorocarbon nanodroplets for drug delivery to cancer. Wiley Interdiscip Rev Nanomed Nanobiotechnol (2012) 4:492–510 10.1002/wnan.1176 22730185 PMC3482424

[37] Chomas JE Dayton PA May D Allen J Klibanov A Ferrara K Optical observation of contrast agent destruction. Appl Phys Lett (2000) 77:1056–8 10.1063/1.1287519

[38] Chomas JE Dayton P May D Ferrara K Threshold of fragmentation for ultrasonic contrast agents. J Biomed Opt (2001) 6:141–50 10.1117/1.1352752 11375723

[39] Chomas JE Dayton P Allen J Morgan K Ferrara KW Mechanisms of contrast agent destruction. IEEE Trans Ultrason Ferroelectr Freq Control (2001) 48:232–48 10.1109/58.896136 11367791

[40] Qin S Caskey CF Ferrara KW Ultrasound contrast microbubbles in imaging and therapy: physical principles and engineering. Phys Med Biol (2009) 54:R27–57 10.1088/0031-9155/54/6/R01 19229096 PMC2818980

[41] Caskey CF Stieger SM Qin S Dayton PA Ferrara KW Direct observations of ultrasound microbubble contrast agent interaction with the microvessel wall. J Acoust Soc Am (2007) 122:1191–200 10.1121/1.2747204 17672665

[42] Caskey CF Qin S Dayton PA Ferrara KW Microbubble tunneling in gel phantoms. J Acoust Soc Am (2009) 125:EL183–9 10.1121/1.3097679 19425620 PMC2736761

[43] Kilroy JP Klibanov AL Wamhoff BR Hossack JA Intravascular ultrasound catheter to enhance microbubble-based drug delivery via acoustic radiation force. IEEE Trans Ultrason Ferroelectr Freq Control (2012) 59:2156–66 10.1109/TUFFC.2012.2442 23143566 PMC4668332

[44] Miller DL Song J Lithotripter shock waves with cavitation nucleation agents produce tumor growth reduction and gene transfer in vivo. Ultrasound Med Biol (2002) 28:1343–8 10.1016/S0301-5629(02)00572-0 12467861

[45] Miller DL Song J Tumor growth reduction and DNA transfer by cavitation-enhanced high-intensity focused ultrasound in vivo. Ultrasound Med Biol (2003) 29:887–93 10.1016/S0301-5629(03)00031-0 12837504

[46] Hauff P Seemann S Reszka R Schultze-Mosgau M Reinhardt M Buzasi T Evaluation of gas-filled microparticles and sonoporation as gene delivery system: feasibility study in rodent tumor models. Radiology (2005) 236:572–8 10.1148/radiol.2362040870 16040915

[47] Howard CM Forsberg F Minimo C Liu JB Merton DA Claudio PP Ultrasound guided site specific gene delivery system using adenoviral vectors and commercial ultrasound contrast agents. J Cell Physiol (2006) 209:413–21 10.1002/jcp.20736 16883597

[48] Sonoda S Tachibana K Uchino E Yamashita T Sakoda K Sonoda KH Inhibition of melanoma by ultrasound-microbubble-aided drug delivery suggests membrane permeabilization. Cancer Biol Ther (2007) 6:1276–83 17704642 10.4161/cbt.6.8.4485

[49] Burke CW Klibanov AL Sheehan JP Price RJ Inhibition of glioma growth by microbubble activation in a subcutaneous model using low duty cycle ultrasound without significant heating. J Neurosurg (2011) 114:1654–61 10.3171/2010.11.JNS101201 21214331 PMC4690208

[50] McDannold N Vykhodtseva N Hynynen K Effects of acoustic parameters and ultrasound contrast agent dose on focused-ultrasound induced blood-brain barrier disruption. Ultrasound Med Biol (2008) 20:20 10.1016/j.ultrasmedbio.2007.11.009 18294757 PMC2459318

[51] Choi JJ Feshitan JA Baseri B Shougang W Yao-Sheng T Borden MA Microbubble-size dependence of focused ultrasound-induced blood-brain barrier opening in mice in vivo. IEEE Trans Biomed Eng (2010) 57:145–54 10.1109/TBME.2009.2034533 19846365 PMC3968777

[52] Stieger SM Caskey CF Adamson RH Qin S Curry FR Wisner ER Enhancement of vascular permeability with low-frequency contrast-enhanced ultrasound in the chorioallantoic membrane model. Radiology (2007) 243:112–21 10.1148/radiol.2431060167 17392250

[53] Delalande A Kotopoulis S Postema M Midoux P Pichon C Sonoporation: mechanistic insights and ongoing challenges for gene transfer. Gene (2013) 525(2):191–9 10.1016/j.gene.2013.03.095 23566843

[54] Sirsi SR Borden MA Advances in ultrasound mediated gene therapy using microbubble contrast agents. Theranostics (2012) 2:1208–22 10.7150/thno.4306 23382777 PMC3563148

[55] Dayton PA Zhao S Bloch SH Schumann P Penrose K Matsunaga TO Application of ultrasound to selectively localize nanodroplets for targeted imaging and therapy. Mol Imaging (2006) 5:160–74 16954031 PMC1752274

[56] Dayton PA Morgan KE Klibanov ALS Brandenburger G Nightingale KR Ferrara KW A preliminary evaluation of the effects of primary and secondary radiation forces on acoustic contrast agents. IEEE Trans Ultrason Ferroelectr Freq Control (1997) 44:1264–77 10.1109/58.656630

[57] Dayton P Klibanov A Brandenburger G Ferrara K Acoustic radiation force in vivo: a mechanism to assist targeting of microbubbles. Ultrasound Med Biol (1999) 25:1195–201 10.1016/S0301-5629(99)00062-9 10576262

[58] O’Reilly MA Hynynen K Ultrasound enhanced drug delivery to the brain and central nervous system. Int J Hyperthermia (2012) 28:386–96 10.3109/02656736.2012.666709 22621739 PMC4078733

[59] Hynynen K McDannold N Martin H Jolesz FA Vykhodtseva N The threshold for brain damage in rabbits induced by bursts of ultrasound in the presence of an ultrasound contrast agent (optison). Ultrasound Med Biol (2003) 29:473–81 10.1016/S0301-5629(02)00741-X 12706199

[60] Baseri B Choi JJ Deffieux T Samiotaki G Tung Y-S Olumolade O Activation of signaling pathways following localized delivery of systemically administered neurotrophic factors across the blood-brain barrier using focused ultrasound and microbubbles. Phys Med Biol (2012) 57:N65–81 10.1088/0031-9155/57/7/N65 22407323 PMC3919955

[61] Choi JJ Feshitan JA Shougang W Yao-Sheng T Baseri B Borden MA The dependence of the ultrasound-induced blood-brain barrier opening characteristics on microbubble size in vivo. AIP Conf Proc (2009) 1113:58–62 10.1063/1.3131471

[62] Choi JJ Small SA Konofagou EE Optimization of blood-brain barrier opening in mice using focused ultrasound. IEEE Ultrasonics Symposium (IEEE Cat. No.06CH37777); 2006 Oct 3–6; Vancouver, Canada (2006). p. 540–3

[63] Konofagou EE Choi J Baseri B Lee A Characterization and optimization of trans-blood-brain barrier diffusion in vivo. AIP Conf Proc (2009) 1113:418–22 10.1063/1.3131462

[64] Konofagou EE Choi J Lee A Baseri B Molecular imaging through the blood-brain barrier: safety assessment and parameter dependence. Proc IEEE Int Symp Biomed Imaging; 2009 Jun 28 to Jul 1; Boston, MA (2009). p. 771–4 10.1109/ISBI.2009.5193163

[65] Konofagou EE Tung Y-S Choi J Deffieux T Baseri B Vlachos F Ultrasound-induced blood-brain barrier opening. Curr Pharm Biotechnol (2012) 13:1332–45 10.2174/138920112800624364 22201586 PMC4038976

[66] Tung YS Marquet F Teichert T Ferrera V Konofagou EE Feasibility of noninvasive cavitation-guided blood-brain barrier opening using focused ultrasound and microbubbles in nonhuman primates. Appl Phys Lett (2011) 98:163704-1–163704-3 10.1063/1.3580763 21580802 PMC3094460

[67] Fite BZ Liu Y Kruse DE Caskey CF Walton JH Lai CY Magnetic resonance thermometry at 7T for real-time monitoring and correction of ultrasound induced mild hyperthermia. PLoS ONE (2012) 7:e35509 10.1371/journal.pone.0035509 22536396 PMC3335017

[68] Lai C Kruse DE Caskey CF Stephens DN Sutcliffe PL Ferrara KW Noninvasive thermometry assisted by a dual-function ultrasound transducer for mild hyperthermia. IEEE Trans Ultrason Ferroelectr Freq Control (2010) 57:2671–84 10.1109/TUFFC.2010.1741 21156363 PMC3018687

[69] Roemer RB Engineering aspects of hyperthermia therapy. Annu Rev Biomed Eng (1999) 1:347–76 10.1146/annurev.bioeng.1.1.347 11701493

[70] Rygh CB Qin SP Seo JW Mahakian LM Zhang H Adamson R Longitudinal investigation of permeability and distribution of macromolecules in mouse malignant transformation using PET. Clin Cancer Res (2011) 17:550–9 10.1158/1078-0432.CCR-10-2049 21106723 PMC3107124

[71] Qin S Seo JW Zhang H Qi J Curry F-RE Ferrara KW An imaging-driven model for liposomal stability and circulation. Mol Pharm (2009) 7:12–21 10.1021/mp900122j 19621944 PMC2867051

[72] Wong AW Ormsby E Zhang H Seo JW Mahakian LM Caskey CF A comparison of image contrast with (64)Cu-labeled long circulating liposomes and (18)F-FDG in a murine model of mammary carcinoma. Am J Nucl Med Mol Imaging (2013) 3:32–43 23342299 PMC3545363

[73] Zheng J Liu J Dunne M Jaffray D Allen C In vivo performance of a liposomal vascular contrast agent for CT and MR-based image guidance applications. Pharm Res (2007) 24:1193–201 10.1007/s11095-006-9220-1 17373581

[74] Huang H Dunne M Lo J Jaffray DA Allen C Comparison of computed tomography- and optical image-based assessment of liposome distribution. Mol Imaging (2013) 12:148–60 23490441

[75] Kim CK Park BK Lee HM Kim SS Kim E MRI techniques for prediction of local tumor progression after high-intensity focused ultrasonic ablation of prostate cancer. AJR Am J Roentgenol (2008) 190:1180–6 10.2214/AJR.07.2924 18430829

[76] Kim CK Park BK Kim B Diffusion-weighted MRI at 3 T for the evaluation of prostate cancer. AJR Am J Roentgenol (2010) 194:1461–9 10.2214/AJR.09.3654 20489084

[77] Hynynen K Damianou C Darkazanli A Unger E Schenck JF The feasibility of using MRI to monitor and guide noninvasive ultrasound surgery. Ultrasound Med Biol (1993) 19:91–2 10.1016/0301-5629(93)90022-G 8456533

[78] Funaki K Fukunishi H Funaki T Sawada K Kaji Y Maruo T Magnetic resonance-guided focused ultrasound surgery for uterine fibroids: relationship between the therapeutic effects and signal intensity of preexisting T2-weighted magnetic resonance images. Am J Obstet Gynecol (2007) 196:e1–6 10.1016/j.ajog.2006.08.030 17306674

[79] McDannold NJ Hynynen K Wolf D Wolf G Jolesz F MRI evaluation of thermal ablation of tumors with focused ultrasound. J Magn Reson Imaging (1998) 8:91–100 10.1002/jmri.1880080119 9500266

[80] Punwani S Emberton M Walkden M Sohaib A Freeman A Ahmed H Prostatic cancer surveillance following whole-gland high-intensity focused ultrasound: comparison of MRI and prostate-specific antigen for detection of residual or recurrent disease. Br J Radiol (2012) 85:720–8 10.1259/bjr/61380797 22253342 PMC3474121

[81] Tempany CM Stewart EA McDannold N Quade BJ Jolesz FA Hynynen K MR imaging-guided focused ultrasound surgery of uterine leiomyomas: a feasibility study. Radiology (2003) 226:897–905 10.1148/radiol.2271020395 12616023

[82] Chen L Bouley D Yuh E D’Arceuil H Butts K Study of focused ultrasound tissue damage using MRI and histology. J Magn Reson Imaging (1999) 10:146–53 10.1002/(SICI)1522-2586(199908)10:2<146::AID-JMRI6>3.3.CO;2-3 10441017

[83] Jacobs MA Ouwerkerk R Kamel I Bottomley PA Bluemke DA Kim HS Proton, diffusion-weighted imaging, and sodium (23Na) MRI of uterine leiomyomata after MR-guided high-intensity focused ultrasound: a preliminary study. J Magn Reson Imaging (2009) 29:649–56 10.1002/jmri.21677 19243047 PMC4151255

[84] Cirillo S Petracchini M D’Urso L Dellamonica P Illing R Regge D Endorectal magnetic resonance imaging and magnetic resonance spectroscopy to monitor the prostate for residual disease or local cancer recurrence after transrectal high-intensity focused ultrasound. BJU Int (2008) 102:452–8 10.1111/j.1464-410X.2008.07633.x 18476973

[85] Wu T Felmlee JP Greenleaf JF Riederer SJ Ehman RL Assessment of thermal tissue ablation with MR elastography. Magn Reson Med (2001) 45:80–7 10.1002/1522-2594(200101)45:1<80::AID-MRM1012>3.0.CO;2-Y 11146489

[86] Kirkham AP Emberton M Hoh IM Illing RO Freeman AA Allen C MR imaging of prostate after treatment with high-intensity focused ultrasound. Radiology (2008) 246:833–44 10.1148/radiol.2463062080 18223121

[87] Rouviere O Lyonnet D Raudrant A Colin-Pangaud C Chapelon JY Bouvier R MRI appearance of prostate following transrectal HIFU ablation of localized cancer. Eur Urol (2001) 40:265–74 10.1159/000049786 11684842

[88] Ben Cheikh A Girouin N Ryon-Taponnier P Mege-Lechevallier F Gelet A Chapelon JY MR detection of local prostate cancer recurrence after transrectal high-intensity focused US treatment: preliminary results. J Radiol (2008) 89:571–7 10.1016/S0221-0363(08)71483-5 18535498

[89] Delli Castelli D Dastru W Terreno E Cittadino E Mainini F Torres E In vivo MRI multicontrast kinetic analysis of the uptake and intracellular trafficking of paramagnetically labeled liposomes. J Control Release (2010) 144:271–9 10.1016/j.jconrel.2010.03.005 20230865

[90] Howseman AM Bowtell RW Functional magnetic resonance imaging: imaging techniques and contrast mechanisms. Philos Trans R Soc Lond B Biol Sci (1999) 354:1179–94 10.1098/rstb.1999.0473 10466145 PMC1692627

[91] Gore JC Manning HC Quarles CC Waddell KW Yankeelov TE Magnetic resonance in the era of molecular imaging of cancer. Magn Reson Imaging (2011) 29:587–600 10.1016/j.mri.2011.02.003 21524870 PMC3285504

[92] Parker DL Applications of NMR imaging in hyperthermia – an evaluation of the potential for localized tissue heating and noninvasive temperature monitoring. IEEE Trans Biomed Eng (1984) 31:161–7 10.1109/TBME.1984.325382 6724602

[93] Rieke V Butts Pauly K MR thermometry. J Magn Reson Imaging (2008) 27:376–90 10.1002/jmri.21265 18219673 PMC2780364

[94] Parker DL Smith V Sheldon P Crooks LE Fussell L Temperature distribution measurements in two-dimensional NMR imaging. Med Phys (1983) 10:321–5 10.1118/1.595307 6877179

[95] Le Bihan D Delannoy J Levin RL Temperature mapping with MR imaging of molecular diffusion: application to hyperthermia. Radiology (1989) 171:853–7 2717764 10.1148/radiology.171.3.2717764

[96] Young IR Hand JW Oatridge A Prior MV Forse GR Further observations on the measurement of tissue T1 to monitor temperature in vivo by MRI. Magn Reson Med (1994) 31:342–5 10.1002/mrm.1910310317 8057809

[97] Graham SJ Stanisz GJ Kecojevic A Bronskill MJ Henkelman RM Analysis of changes in MR properties of tissues after heat treatment. Magn Reson Med (1999) 42:1061–71 10.1002/(SICI)1522-2594(199912)42:6<1061::AID-MRM10>3.0.CO;2-T 10571927

[98] Chen J Daniel BL Pauly KB Investigation of proton density for measuring tissue temperature. J Magn Reson Imaging (2006) 23:430–7 10.1002/jmri.20516 16463298

[99] Ishihara Y Calderon A Watanabe H Okamoto K Suzuki Y Kuroda K Precise and fast temperature mapping using water proton chemical-shift. Magn Reson Med (1995) 34:814–23 10.1002/mrm.1910340606 8598808

[100] Galiana G Branca RT Jenista ER Warren WS Accurate temperature imaging based on intermolecular coherences in magnetic resonance. Science (2008) 322:421–4 10.1126/science.1163242 18927389 PMC3080759

[101] Quesson B de Zwart JA Moonen CTW Magnetic resonance temperature imaging for guidance of thermotherapy. J Magn Reson Imaging (2000) 12:525–33 10.1002/1522-2586(200010)12:4<525::AID-JMRI3>3.0.CO;2-V 11042633

[102] Peters RD Hinks RS Henkelman RM Ex vivo tissue-type independence in proton-resonance frequency shift MR thermometry. Magn Reson Med (1998) 40:454–9 10.1002/mrm.1910400316 9727949

[103] Weidensteiner C Kerioui N Quesson B de Senneville BD Trillaud H Moonen CT Stability of real-time MR temperature mapping in healthy and diseased human liver. J Magn Reson Imaging (2004) 19:438–46 10.1002/jmri.20019 15065167

[104] Bankson JA Stafford RJ Hazle JD Partially parallel imaging with phase-sensitive data: increased temporal resolution for magnetic resonance temperature imaging. Magn Reson Med (2005) 53:658–65 10.1002/mrm.20378 15723414

[105] Guo JY Kholmovski EG Zhang L Jeong EK Parker DL k-Space inherited parallel acquisition (KIPA): application on dynamic magnetic resonance imaging thermometry. Magn Reson Imaging (2006) 24:903–15 10.1016/j.mri.2006.03.001 16916708

[106] Kohler MO Mougenot C Quesson B Enholm J Le Bail B Laurent C Volumetric HIFU ablation under 3D guidance of rapid MRI thermometry. Med Phys (2009) 36:3521–35 10.1118/1.3152112 19746786

[107] Enholm JK Kohler MO Quesson B Mougenot C Moonen CTW Sokka SD Improved volumetric MR-HIFU ablation by robust binary feedback control. IEEE Trans Biomed Eng (2010) 57:103–13 10.1109/TBME.2009.2034636 19846364

[108] Ergun AS Analytical and numerical calculations of optimum design frequency for focused ultrasound therapy and acoustic radiation force. Ultrasonics (2011) 51:786–94 10.1016/j.ultras.2011.03.006 21459399

[109] Wu F Wang Z-B Cao Y-D Zhou Q Zhang Y Xu Z-L Expression of tumor antigens and heat-shock protein 70 in breast cancer cells after high-intensity focused ultrasound ablation. Ann Surg Oncol (2007) 14:1237–42 10.1245/s10434-006-9275-6 17187168

[110] Wu F Zhou L Chen WR Host antitumour immune responses to HIFU ablation. Int J Hyperthermia (2007) 23:165–71 10.1080/02656730701206638 17578340

[111] Zhou Q Zhu X-Q Zhang J Xu Z-L Lu P Wu F Changes in circulating immunosuppressive cytokine levels of cancer patients after high intensity focused ultrasound treatment. Ultrasound Med Biol (2008) 34:81–7 10.1016/j.ultrasmedbio.2007.07.013 17854983

[112] Lu P Zhu X-Q Xu Z-L Zhou Q Zhang J Wu F Increased infiltration of activated tumor-infiltrating lymphocytes after high intensity focused ultrasound ablation of human breast cancer. Surgery (2009) 145:286–93 10.1016/j.surg.2008.10.010 19231581

[113] Xu Z-L Zhu X-Q Lu P Zhou Q Zhang J Wu F Activation of tumor-infiltrating antigen presenting cells by high intensity focused ultrasound ablation of human breast cancer. Ultrasound Med Biol (2009) 35:50–7 10.1016/j.ultrasmedbio.2008.08.005 18950932

[114] Zhang Y Deng J Feng J Wu F Enhancement of antitumor vaccine in ablated hepatocellular carcinoma by high-intensity focused ultrasound. World J Gastroenterol (2010) 16:3584–91 10.3748/wjg.v16.i28.3584 20653069 PMC2909560

[115] Deng J Zhang Y Feng J Wu F Dendritic cells loaded with ultrasound-ablated tumor induce in vivo specific antitumor responses. Ultrasound Med Biol (2010) 36:441–8 10.1016/j.ultrasmedbio.2009.12.004 20172447

[116] Higgins JP Bernstein MB Hodge JW Enhancing immune responses to tumor-associated antigens. Cancer Biol Ther (2009) 8:1440–9 10.4161/cbt.8.15.9133 19556848 PMC7236598

[117] Liu F Hu Z Qiu L Hui C Li C Zhong P Boosting high-intensity focused ultrasound-induced anti-tumor immunity using a sparse-scan strategy that can more effectively promote dendritic cell maturation. J Transl Med (2010) 8:7 10.1186/1479-5876-8-7 20105334 PMC2842246

[118] Dalfior D Delahunt B Brunelli M Parisi A Ficarra V Novara G Utility of racemase and other immunomarkers in the detection of adenocarcinoma in prostatic tissue damaged by high intensity focused ultrasound therapy. Pathology (2010) 42:1–5 10.3109/00313020903434447 20025473

[119] Zhong-Lin X Xue-Qiang Z Pei L Qiang Z Jun Z Feng W Activation of tumor-infiltrating antigen presenting cells by high intensity focused ultrasound ablation of human breast cancer. Ultrasound Med Biol (2009) 35(1):50–7 10.1016/j.ultrasmedbio.2008.08.005 18950932

[120] Zhang H Liu L Sharma R Alfieri A Saha S Garg M High intensity focused ultrasound (HIFU)-enhanced autologous in situ vaccination for prostate cancer. Int J Radiat Oncol Biol Phys (2008) 72:S165–6 10.1016/j.ijrobp.2008.06.516

[121] Hu Z Yang XY Liu Y Sankin GN Pua EC Morse MA Investigation of HIFU-induced anti-tumor immunity in a murine tumor model. J Transl Med (2007) 5:34 10.1186/1479-5876-5-34 17625013 PMC1939831

[122] Zerbini A Pilli M Fagnoni F Pelosi G Pizzi MG Schivazappa S Increased immunostimulatory activity conferred to antigen-presenting cells by exposure to antigen extract from hepatocellular carcinoma after radiofrequency thermal ablation. J Immunother (2008) 31:271–82 10.1097/CJI.0b013e318160ff1c 18317360

[123] ter Haar G Rivens I Chen L Riddler S High intensity focused ultrasound for the treatment of rat tumours. Phys Med Biol (1991) 36:1495–501 10.1088/0031-9155/36/11/009 1754620

[124] Chen L ter Haar G Hill CR Eccles SA Box G Treatment of implanted liver tumors with focused ultrasound. Ultrasound Med Biol (1998) 24:1475–88 10.1016/S0301-5629(98)00134-3 10385969

